# The Effects of Silver Nanoparticles (AgNPs) on Thermophilic Bacteria: Antibacterial, Morphological, Physiological and Biochemical Investigations

**DOI:** 10.3390/microorganisms12020402

**Published:** 2024-02-17

**Authors:** Israt Jahan, Fatma Matpan Bekler, Ahmed Tunç, Kemal Güven

**Affiliations:** 1 Department of Health Care Services, Vocational School of Health Services, Mardin Artuklu University, 47100 Mardin, Türkiye; sonia.israt2@gmail.com; 2 Department of Molecular Biology and Genetics, Faculty of Science, Dicle University, 21280 Diyarbakir, Türkiye; fmatpan@dicle.edu.tr; 3 Department of Interdisciplinary Nanotechnology, Graduate School of Natural and Applied Sciences, Dicle University, 21280 Diyarbakir, Türkiye; ahmet_tunc@hotmail.com

**Keywords:** silver nanoparticles, antibacterial toxicity, thermophilic bacteria, β-galactosidase inhibition, superoxide radicals (SORs), TEM, FT-IR

## Abstract

Since thermophilic microorganisms are valuable sources of thermostable enzymes, it is essential to recognize the potential toxicity of silver nanoparticles used in diverse industrial sectors. Thermophilic bacteria *Geobacillus vulcani* 2Cx, *Bacillus licheniformis* 3CA, *Paenibacillus macerans* 3CA1, *Anoxybacillus ayderensis* FMB1, and *Bacillus paralicheniformis* FMB2-1 were selected, and their MIC and MBC values were assessed by treatment with AgNPs in a range of 62.5–1500 μg mL^−1^. The growth inhibition curves showed that the *G. vulcani* 2Cx, and *B. paralicheniformis* FMB2-1 strains were more sensitive to AgNPs, demonstrating a reduction in population by 71.1% and 31.7% at 62.5 μg mL^−1^ and by 82.9% and 72.8% at 250 μg mL^−1^, respectively. TEM and FT-IR analysis revealed that AgNPs caused structural damage, cytoplasmic leakage, and disruption of cellular integrity. Furthermore, cell viability showed a significant decrease alongside an increase in superoxide radical (SOR; O_2_^−^) production. β-galactosidase biosynthesis decreased to 28.8% level at 500 μg mL^−1^ AgNPs for *G. vulcani* 2Cx, 32.2% at 250 μg mL^−1^ for *A. ayderensis* FMB1, and 38.8% only at 62.5 μg mL^−1^, but it was completely inhibited at 500 μg mL^−1^ for *B. licheniformis* 3CA. Moreover, *B. paralicheniformis* FMB2-1 showed a significant decrease to 11.2% at 125 μg mL^−1^. This study is the first to reveal the toxic effects of AgNPs on thermophilic bacteria.

## 1. Introduction

Over the past two decades, there has been a notable surge in industrial engagement with nanoscience and the manufacturing of nanotech-integrated items [1]. Within the United States, for instance, a significant proportion (94%) of nano-scaled components has been discharged into the natural ecosystem through the application of cosmetics and personal grooming items, which predominantly comprises silver (1.2–272 t/year), titanium dioxide (870–1000 t/year), and zinc oxide (1800–2100 t/year) [2,3]. The excessive generation, application, and improper handling of nano-sized substances have expedited their release into multiple ecological domains, which may lead to potential environmental contaminations. Hence, ongoing research continues to explore the potential implications of nanoparticles on both human health and the ecosystem, underscoring their potential impact. [4] The term “nanoparticle” refers to a comprehensive range of elements that encompass particulate substances with the lowest extent of one dimension measuring less than 100 nm [5] They have found applications across diverse fields spanning medicine, biological science, electronics, agricultural and environmental science, and energy [6]. Their small size facilitates the penetration of biological barriers and targeted delivery to specific sites within the body, thereby enhancing the efficiency and effectiveness of therapeutic or other biological interventions [7].

Compared with other nanomaterials, silver nanoparticles (AgNPs) have received substantial recognition because of their distinctive properties [8]. The reason they especially have gained so much attention is because AgNPs have found widespread application across diverse fields such as in household, industrial, and consumer goods; cosmetics; textiles; food processing and packaging; pharmaceuticals; medical devices; diagnostics; orthopedics; drug delivery; wound dressings; and the development of antibacterial and anticancer agents [9,10]. They have also been applied in preventing biofilm formation and inhibiting the growth of pathogens on catheters, cardiovascular implants, and bone implants, thereby offering promising avenues for treatment [11,12]. As the utilization of AgNPs continues to expand, it becomes increasingly imperative to acquire a deeper comprehension of their toxicity and the underlying mechanisms involved.

Nanoparticles (NPs) are increasingly used to target both Gram-negative and Gram-positive bacteria either alone or in combination with antibiotics to combat multidrug resistance in pathogenic bacteria due to broad-spectrum antibacterial properties [13]. The antibacterial impact of AgNPs is considered to be due to their smaller particle size, which has an efficient penetration ability into bacterial cells, particularly into Gram-negative ones [14]. The antibacterial effect of AgNPs is also concentration-dependent [15]. In general, it is well studied that the size of nanoparticles should be smaller than 50 nm to be effective for enhanced antimicrobial activity [16]. In addition, the shapes of the nanoparticles also show different effects on interactions, so it has been reported that spherical nanoparticles with a larger effective specific contact area cause more damage than rod-shaped or wire-shaped nanoparticles by making closer contact with the bacterial cell [17,18].

Many studies carried out recently have revealed that microbial activity rates result in a decrease in microbial population and diversity, particularly in the soil microbial biomass, including nitrogen-fixing and ammonia-oxidizing microorganisms [19,20,21,22,23,24]. While certain studies have posited that the toxicity of AgNPs primarily stems from the release of Ag^+^ ions that readily infiltrate bacterial cells resulting in cellular damage and the inhibition of essential functions, other factors may also contribute to the observed toxicity [25]. Metal ions, when released gradually from NPs and absorbed onto the cell membrane, directly interact with functional groups (such as mercapto, amino, and carboxyl groups) found in nucleic acids and proteins. These interactions lead to enzyme activity impairment, changes in cell structure, disruptions in normal physiological processes, and ultimately the inhibition of microorganisms [26]. Moreover, numerous studies have consistently demonstrated that the toxicity of AgNPs primarily stems from their direct interaction with functional groups present on the cell surface, which is subsequently followed by internalization into the cells. This interaction causes detrimental effects such as membrane damage, oxidative stress, and significant mortality [14,27].

Extensive research has been documented in the literature exploring the effects of diverse nanoparticles (NPs) on enzyme activity in microorganisms, animals, and plants [28,29,30,31,32,33]. In particular, biochemical, physiological, and molecular effects have been investigated in different soil organisms and microorganisms. AgNPs have been demonstrated to inhibit the activity of numerous extracellular enzymes, such as urease, phosphatase, phosphomonoesterase, β-D-glucosidase, leucine-aminopeptidase, and arylsulfatase. This inhibition can be attributed to the binding of the released Ag+ ions to the thiol groups of the enzymes or the direct interaction between AgNPs and the enzymes, potentially altering their conformation or obstructing the active site [27,34]. Unlike certain small molecules and biological molecules, metallic nanoparticles (NPs) exhibit a notable propensity for easy cellular entry [35]. Additionally, metallic nanoparticles (NPs) engage with essential components within bacterial cells, such as ribosomes, enzymes, and DNA, inducing protein deactivation, enzyme inhibition, and modulation of gene expression [36,37].

Thermophilic bacteria primarily thrive in hot springs, enduring and adapting to temperatures ranging from 40 to 120 °C. They possess physically and chemically stable enzymes with unique macromolecular properties, allowing them to thrive at high temperatures and achieve higher end product yields compared with those of mesophilic bacteria. Thermophilic microorganisms have raised special interest and demand over the past few years as a source of novel, thermostable enzymes that have applications particularly in the sugar industry and starch processing; production of low-lactose milk; alcohol production; the fruit, paper, and leather industries; and in laundry detergents [38,39,40,41,42]. The unique structure of the cell wall and the mechanisms of their adaptation also make thermophilics appropriate candidates for the bioremediation of metals from different environments or for the remediation of textile dyes [42,43,44,45,46]. The *Bacillus* genus and its respective species are highly valued in the enzyme and pharmaceutical industries thanks to the significant presence of potential bioactive compounds that they have [12]. As thermophilic microorganisms play a crucial role in the production of thermostable enzymes like β-galactosidase [47,48,49,50,51], there is a lack of a comprehensive understanding regarding the antibacterial mechanisms of AgNPs and the factors that impact enzyme biosynthesis, secretion, and inhibition. Thus, a detailed elucidation of these aspects is essential.

Therefore, this study is specifically designed to explore the effects of different concentrations of AgNPs on the structural integrity and vital functions of thermophilic bacteria, and this includes (i) assessing the susceptibility of bacterial strains and their growth under AgNP-induced stress, (ii) evaluating changes in cell morphology, (iii) detecting AgNP interaction with bacterial biomasses through FT-IR analysis, (iv) examining toxic effects on cellular respiration, (v) measuring superoxide production, and (vi) investigating the inhibition of enzyme biosynthesis and extracellular secretion under AgNP-induced stress.

## 2. Materials and Methods

### 2.1. Silver Nanoparticles (AgNPs)

AgNPs were obtained commercially from chemPUR (Karlsruhe, Germany) and their physicochemical characteristics are shown in Table 1.

### 2.2. Determination of Silver (Ag) Ion Release from AgNPs

To determine the effect of the utilized basal medium on 250 μg mL^−1^ concentrations of AgNPs, the samples were kept on a shaking incubator (at 100 rpm for 24 h), and metal ion release from AgNPs was measured by FAAS (PinAAcle 500 Flame Atomic Absorption Spectrometer, PerkinElmer, New Castle, DE, USA) following the modified method described by Dong et al. [52].

### 2.3. Strains and Maintenance of Cultures

The strains used in the present study were Geobacillus *vulcani* 2Cx (GenBank accesion number: MT350132), *Bacillus licheniformis* 3CA (GenBank accesion number: MT350128), and *Paenibacillus macerans* 3CA1 (GenBank accesion number: MT350131), which were isolated and identified from the Diyarbakır Çermik hot water spring, (38°8′27.2544″ N, 39°28′46.6068″ E) [53], *Anoxybacillus ayderensis* FMB1 (GenBank accesion number: KP992869) [54] and *Bacillus paralicheniformis* FMB2-1 (GenBank accesion number: KP992870) [55], which were isolated and identified from the Yozgat Sorgun hot water spring (39°48′14.0718″ N, 35°12′31.0752″ E).

All stock bacterial strains were first introduced into Nutrient Broth (NB), which served as the growth medium. The inoculated bacterial cultures were placed in a water bath shaker set at 50 °C for a duration between 12 and 24 h to allow the bacteria to grow. After the incubation period, the bacterial cultures were subjected to centrifugation at room temperature and a speed of 10,000 rpm for 10 min. The resulting centrifuged mixture yielded a supernatant and a bacterial pellet. The supernatant, which contained liquid and other cellular debris, was carefully discarded. The bacterial pellet, which consisted of concentrated bacterial cells, was retained and collected for further analysis or experimentation. For AgNP treatment experiments, bacterial cultures (1 × 10^7^ CFU mL^−1^) were inoculated into the amended basal medium (BM: 0.4 g L^−1^ yeast extract, 1 g L^−1^ peptone, 1 g L^−1^ sodium chloride) and on a basal medium agar (BMA: BM: 0.4 g L^−1^ yeast extract, 1 g L^−1^ peptone, 1 g L^−1^ sodium chloride and agar 15 g L^−1^) at a 1% inoculum rate. A control group was included for each bacterial strain where no AgNPs were introduced. To account for the potential influence of incident light reflectance by nanoparticles (NPs), negative controls consisting solely of AgNPs were included during the exposures. The absorbance values of these negative controls were then utilized to subtract any fluctuations observed in the inoculated cultures. All treatments were replicated at least three times.

### 2.4. Susceptibility of Bacterial Strains to Ag Nanoparticles

Strains of *G. vulcani* 2Cx, *B. licheniformis* 3CA, *P. macerans* 3CA1, *A. ayderensis* FMB1, and *B. paralicheniformis* FMB2-1 were cultivated in the BM at 50 °C, at pH 7.0, and at optimal conditions.

In order to determine the AgNPs’ sensitivities/resistances of these bacterial strains, each strain was grown separately in a liquid BM under optimum growth conditions, and a 12 h overnight culture was obtained. The BM medium and various concentrations (62.5, 125, 250, 500, 1000, and 1500 μg mL^−1^) of AgNPs were added to 15 mL sterile tubes and inoculated with an equal amount of bacteria (0.1 mL) from the 12 h fresh culture and incubated at 50 °C for 24 h in a shaking incubator (at 100 rpm). AgNPs in different concentrations in which bacteria were not added were used as the negative control, and the medium in which bacteria were grown without AgNPs was used as the positive control. At the end of the incubation, all samples tested for growth inhibition of bacterial cells by AgNPs were measured at 600 nm in the spectrophotometer (Libra Biochrom, Cambridge, United Kingdom), and the absorbance values were obtained. The minimum inhibitory concentration (MIC) was determined as the lowest concentration of AgNPs that effectively inhibited bacterial growth by 99%. Cell viability was quantified by counting the number of colony-forming units (CFUs) per milliliter, and the calculation was performed using the following formula:

$$CFU\;{mL}^{-1}=\frac{\left(number\;of\;colonies\times dilution\;factor\right)}{volume\;covered\;(mL)}$$

Bacteria were uniformly spread from 0.1 mL of the bacterial cultures into a semi-solid BM agar medium and incubated as mentioned above. The MBC representing the lowest AgNP concentration at which all bacterial cells were completely eliminated in the medium was determined.

### 2.5. Exploring Cellular Damage through Transmission Electron Microscopy (TEM)

All bacterial strains, with the exception of the control group, were exposed to the prepared AgNP concentrations specified as 62.5, 125, 250, or 500 μg mL^−1^. The bacterial cultures were exposed to AgNPs and were allowed to incubate under favorable growth conditions for 24 h. After the incubation period, the bacterial cultures were subjected to centrifugation at room temperature at a speed of 10,000 rpm for 10 min. The supernatant, which contained liquid and other cellular debris, was carefully discarded and repeated the centrifuge cycle several times by adding sterile phosphate-buffered saline (PBS 1x) with a concentration of 10 mM and a pH of 7.2. The cellular damage caused by AgNPs was observed using a Jeol brand (model JEM-1010, Cambridge, MA, USA) Transmission Electron Microscope (TEM), equipped with a GATAN brand 782 ES500W camera system (Biorad, CA, USA). The speed voltage was increased to 120 kV to facilitate the observation process. To prepare the specimens for TEM analysis, a low amount of each cell suspension was carefully placed drop by drop onto copper grids. Subsequently, the grids were left to dry naturally at room temperature for a few hours. Once dried, the prepared specimens were ready for TEM imaging.

### 2.6. FT-IR Analysis of Bacterial Biomass Treated with Nanoparticles

Fourier transform infrared spectroscopy (FT-IR) analysis was conducted to qualitatively assess the surface functional groups of the materials. For this, all bacterial strains were grown overnight in a BM medium at 50 °C. Following washing and centrifugation, each strain sample was supplemented with a final concentration of 500 μg mL^−1^ of AgNPs while ensuring a cell density of 10^8−9^ CFU mL^−1^ was maintained. After incubating for 24 h, the bacterial cultures, both treated with AgNPs and untreated, were subjected to centrifugation to separate the pellet. The resulting biomass was then dried at 60 °C under vacuum conditions in a vacuum incubator until it reached a weight of 2.5 mg. The biomass samples obtained were recorded in the FT-IR spectrophotometer device (Perkin Elmer Spectrum 100, New Castle, DE, USA) in the reduced total reflection (ATR) system with a resolution of 16 cm^−1^ and a wavenumber range of 4000 to 450 cm^−1^ subjected to 20 scans.

### 2.7. Evaluating Cellular Viability in the Presence of NP Stress

All bacterial strains, except the control group, were subjected to treatment with the prepared AgNPs (62.5, 125, 250, or 500 μg mL^−1^). The bacterial cultures were exposed to the AgNPs and allowed to incubate under favorable growth conditions for 12 h. After the incubation period, the bacterial cultures were assessed for cell viability.

Cell viability was evaluated using the MTT assay. PBS with a concentration of 10 mM and a pH of 7.2 was used as solvent to prepare a stock solution of 5 mg mL^−1^ of MTT 3-(4,5-Dimethyl-2-thiazolyl)-2,5-diphenyl-2H-tetrazolium bromide). The solution was then sterilized through filtration and were carefully maintained and stored at a precise temperature of −20 °C for future applications. For each treatment, including the control, a volume of 500 µL of the cell suspension was carefully transferred to individual 1.5 mL microcentrifuge tubes. Subsequently, 50 µL of the MTT solution was integrated in each tube, and proper mixing was ensured by vortexing. The tubes were then placed in a water bath shaker at a temperature of 50 °C and allowed to incubate for a duration of 1 h. After the incubation period, the bacterial cultures underwent centrifugation at room temperature at a speed of 12.000 rpm for 10 min to collect the pellet. The supernatant was thrown away, and 500 µL of 100% dimethyl sulfoxide (DMSO) was added to each tube. The tubes were incubated again at 30 °C for 30 min to dissolve crystalized formazan. Subsequently, a volume of 200 µL of the supernatant, which exhibited a distinctive purple color, was carefully transferred to the individual wells of a 96-well plate. Every single treatment was replicated twice, and blanks (PBS only) were included as the negative controls for each group. The optical density of the samples was determined using a microplate reader (Multi ScanGo, Thermo Fisher, Vantaa, Finland) at a 570 nm wavelength. The cell viability percentage was ascertained by employing the provided following formula, which relies on absorbance values:

(1) $$\%\;\mathrm{viability}\;=\;\frac{Absorbance\;of\;treated\;cells\;}{Absorbance\;of\;untreated/control\;cells}\;\;\;\;\%100$$

### 2.8. Quantifying Superoxide Generation in Bacterial Cells under NP-Induced Stress

The bacterial cultures were exposed to AgNPs to assess the generation of superoxide anions, as described in previous Section 2.7. To prepare a stock solution of nitroblue tetrazolium (NBT), 8 mg mL^−1^ of NBT powder was meticulously dissolved in sterile ultra-purified water. The solution was then stored in a refrigerator at 4 °C for future use. For each treatment followed by 12 h incubation, including the control group, a precise volume of 200 µL of the cell suspension was meticulously transferred to the individual wells of a 96-well plate. Each treatment was replicated twice to ensure reliability, and blanks consisting of phosphate-buffered saline (PBS) alone were included as the negative controls for each group. Subsequently, a precise volume of 20 µL of the NBT solution was carefully added to each well containing the cell suspension. The plate was then incubated at a temperature of 50 °C with continuous shaking for a duration of 2 h. During this incubation period, SORs released by the cells reacted with NBT, forming blue-colored formazan deposits. After the incubation period, the absorbance of the cell suspensions was measured at a wavelength of 570 nm using a microplate reader (Multi ScanGo, Thermo). This measurement allowed for the quantification of the deposited formazan and served as an indicator of superoxide anion generation by the cells.

### 2.9. Effect of Silver NPs on Enzyme Biosynthesis

In order to determine the β-galactosidase biosynthesis by bacterial strains, the liquid BM containing 2% lactose was prepared by adding several AgNP concentrations (62.5, 125, 250, or 500 μg mL^−1^) and inoculated with the bacterial strains for 24 h in a shaking incubator at 50 °C, and a 650 µL portion of the culture sample was combined with an equal volume of 0.1 M sodium phosphate buffer (pH 7.0) to facilitate cell permeabilization. Subsequently, 0.01% sodium dodecyl sulfate (SDS) was added to the mixture (as described in [56,57]) and incubated at 50 °C for 10 min. Following that, a solution of 60 mM o-nitrophenyl-β-D-galactopyranoside (oNPG) was introduced to the mixture and allowed to incubate at 50 °C for an additional 10 min. The reaction was subsequently terminated by the addition of Na_2_CO_3_. Enzyme activity was measured in a spectrophotometer (Libra Biochrom, Cambridge, United Kingdom) at 405 nm. The residual activity in the absence of AgNPs for all strains (controls) was taken as 100%. All data were the mean values of at least 3 experiments.

### 2.10. Effect of Silver NPs on Enzyme Secretion and Inhibition

Bacterial cultures were grown in BM broth supplemented with 2% lactose. When the bacterial cells reached the early exponential growth phase, they were separated by centrifugation at 10,000 rpm for 10 min. The resulting pellets were resuspended in a solution of 0.02 M sodium phosphate buffer (pH 7.5) containing 0.01 M NaCl. Cell density was maintained at approximately 10^8−9^ CFU mL^−1^. For each strain, 500 µL of cell suspension was treated with 250 µg mL^−1^ AgNPs along with non-treated controls containing only bacterial strains. The mixture was incubated for 4 h at 50 °C in a shaking incubator (100 rpm). After the completion of the incubation period, 500 µL of a 0.02 M sodium phosphate buffer (pH 7.5) containing 0.01 M NaCl was added to both the control and treated cell suspensions. The supernatants obtained by centrifuging the suspensions at 12,000 rpm for 10 min were used to assess the extracellular ß-galactosidase activity. The activity was measured at 405 nm using *o*NPG as the substrate on a spectrophotometer. All data were the mean values of at least 3 experiments.

Bacterial strainsl, namely *A. ayderensis* FMB1 and *B. licheniformis* 3CA, that extracellularly secrete the enzyme more efficiently compared withothers were also utilized in order to determine whether various AgNP concentrations inhibit the activity of ß-galactosidases. The cultures were produced under optimum conditions in a AgNP-free liquid BM, and supernatants were obtained by centrifugation. Then, 500 μL 0.02 M of sodium phosphate buffer (pH 7.5) containing AgNPs (final concentrations between 62.5 to 250 μg mL^−1^) was added to 500 μL of each supernatant and incubated at 50 °C for 30 min for the interaction of the nanoparticle and enzyme. At the end of this period, the reaction was carried out by adding *o*NPG and incubated at 50 °C for 10 min. The enzyme activity was measured spectrophotometrically at 405 nm. The crude enzyme samples without any added AgNPs were used as the control (the relative activities were considered to be 100%), and the relative activities were determined by calculating the average of at least three replicates.

### 2.11. Statistical Analysis

The data obtained from the experiment underwent statistical analysis utilizing one-way analysis of variance (ANOVA) with the significance level set at 5%. The least significant difference (LSD) was computed as a measure to assess the variation between the means of different treatments. In addition, for comparing the differences among the treatment means, Duncan’s multiple range test (DMRT) was utilized at a significance level of 5%. The data depicted in the figures were represented as the mean ± standard deviation (SD) and were based on two independent replicates conducted for each measured parameter.

## 3. Results and Discussion

### 3.1. Determination of Silver (Ag) Ion Release from AgNPs

To explore the extent of the ionic silver dissolution from AgNPs, which may cause cellular toxicity, AgNPs at 250 μg mL^−1^ were evaluated after 24 h incubation. The calculated dissolved silver (Ag) was found to be as low as 0.55% in the basal medium at 50 °C.

The inclusion of silver nanoparticles in a wide range of manufactured goods contributes to their discharge into aquatic ecosystem, leading to the presence of dissolved silver (Ag^+^) subsequently exerting toxic impacts on diverse aquatic life-forms, including bacteria, algae, fish, and daphnia [58]. NPs can easily enter cellular entities through inhalation, ingestion, or skin absorption due to their nano-scale size. This property allows them to have significant interactions within human and other living organisms [59]. There have been ongoing debates about how Ag^+^ released from AgNPs is the main factor in cellular toxicity as it readily enters the cells, inhibiting several vital functions [24]. Metal ions slowly released from NPs interact with the functional groups of biomolecules, inhibiting enzyme activities, altering the cell structure, interfering with the normal physiological processes, and finally affecting the microorganism [26]. On the other hand, the toxic effects of AgNPs may probably be due to AgNP itself binding to biomolecules, thereby leading to the inhibition of protein biosynthesis and enzyme activity [27,34].

The bactericidal actions of the silver ion itself determined by the MICs and MBCs against various pathogens were investigated, and the MICs and MBCs of silver ion were found to range from 1.9 to 15.6 µg/mL [60]. Moreover, the MICs and MBCs of ionic silver and chemically produced nanosilver were compared in the growth medium for three bacterial species, namely *E. coli*, *P. aeruginosa*, and *S. aureus*, after 24 h of exposure. For all three bacteria, the MIC and MBC values for nanosilver were 500 µg/mL or above, while they ranged between 12.5 and 200 µg/mL for ionic silver [61]. From this point of view, it seems that antibacterial effects of silver ions are seen at rather high concentrations, and the extremely low Ag^+^ release from AgNPs (0.55%) could not be thus considered to be the only cause of toxicity on the thermophilic bacteria. Zhang et al. [62] also found that the amount of Ag^+^ released from AgNPs was less than 0.5% and discussed how the partial reduction in toxicity for mesophilic bacteria may be attributed to a decrease in the release of silver ions.

### 3.2. Phenotypic Characterization of Bacterial Strains

The strains of *G. vulcani* 2Cx, *B. licheniformis* 3CA, *P. macerans* 3CA1, *A. ayderensis* FMB1, and *B. paralicheniformis* FMB2-1 were Gram-positive and rod-shaped. Bacterial strains having varying morphological and physiological features are shown in Table 2.

### 3.3. Bacterial Growth and NP Tolerance/Sensitivity

The strains of *G. vulcani* 2Cx, *B. licheniformis* 3CA, *P. macerans* 3CA1, *A. ayderensis* FMB1, and *B. paralicheniformis* FMB2-1 grown in a specific basal medium exposed to various AgNP concentrations (62.5–1500 μg mL^−1^) showed differential resistance/sensitivity behavior. The MIC and MBC of AgNPs against *G. vulcani* 2Cx, *B. licheniformis* 3CA, *P. macerans* 3CA1, *A. ayderensis* FMB1, and *B. paralicheniformis* FMB2-1 are shown in Table 3.

**Figure 1:**
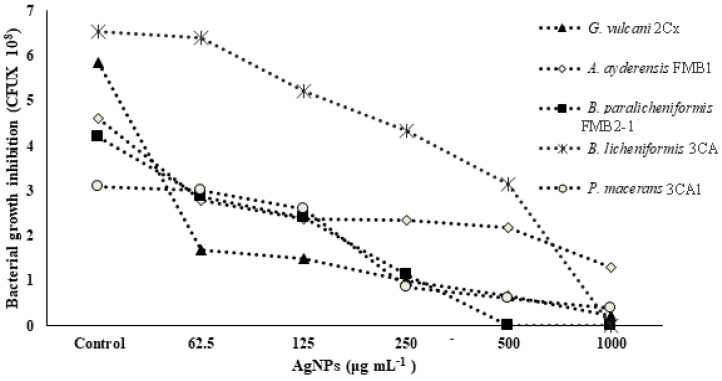
Concentration-dependent growth inhibition of bacterial cells by AgNPs. All samples were measured at 600 nm in the spectrophotometer, and the absorbance values were converted to colony forming units.

In Table 3, the MIC (minimum inhibitory concentration) and MBC (minimum bactericidal concentration) values of AgNPs against five different strains of thermophilic bacteria are presented. The highest concentration of AgNPs (1000 or 1500 μg mL^−1^) was found to be ineffective in determining the MIC and MBC values for certain strains of thermophilic bacteria. The results demonstrated a significant reduction in cell numbers at the lowest levels for the corresponding MIC, and a complete loss was observed for the MBC. Figure 1 shows the growth inhibition of thermophilic bacteria strains depending on the increasing concentrations of AgNPs. It is evident that the strains *G. vulcani* 2Cx and *B. paralicheniformis* FMB2-1 exhibit higher sensitivity to AgNP treatments. There was a sharp growth inhibition (71.1%) for *G. vulcani* 2Cx in the presence of AgNPs even at 62.5 μg mL^−1^ compared with that of the control, while growth was decreased slowly between the 62.5 and 1000 μg mL^−1^ AgNP concentrations. Moreover, *B. paralicheniformis* FMB2-1 growth also appears to be inhibited sharply between the 62.5 μg mL^−1^ and 500 μg mL^−1^ AgNP concentrations. In contrast, the growth inhibition of *P. macerans* 3CA1 is minimal at AgNP concentrations of 62.5 and 125 μg mL^−1^, with only 2.6% and 15.6% inhibition, respectively. However, a pronounced inhibition of 72.5% is observed for the concentration of 250 μg mL^−1^. However, it can also be seen that growth inhibition for *A. ayderensis* FMB1 and *B. licheniformis* 3CA are decreased consistently at AgNP concentrations starting from 62.5 to 1000 μg mL^−1^.

The antibacterial effect of AgNPs is attributed to their small particle size, which allows for excellent penetration into bacteria, especially Gram-negative bacteria. Furthermore, this effect is known to be concentration-dependent, meaning that higher concentrations of AgNPs result in increased antibacterial activity [14,15]. The growth inhibition for *S. aureus* was found to be less remarkable (MIC value of 33 nM), while even low AgNP concentrations inhibited *E. coli* growth (MIC of 3.3 nM) considerably [11]. Ahmed et al. [63] also studied the impact of metal NPs on the growth behavior of soil bacteria such as *S. meliloti*, *P. mosselii*, *A. chroococcum*, and *B. thuringiensis*, which revealed that all sub-MICs of metal NPs delayed the bacterial growth for all test strains, while higher concentrations totally abolished the growth. In the case of AgNPs, the MIC concentrations were observed as 1000, 500, 250, and 500 µg mL^−1^, while the MBC concentrations were 1500, 1000, 500, and 1000 µg mL^−1^ for *B. thuringiensis*, *P. mosselii*, *S. meliloti,* and *A. chroococcum,* respectively.

### 3.4. Exploring Cellular Damage through Transmission Electron Microscopy (TEM)

**Figure 2:**
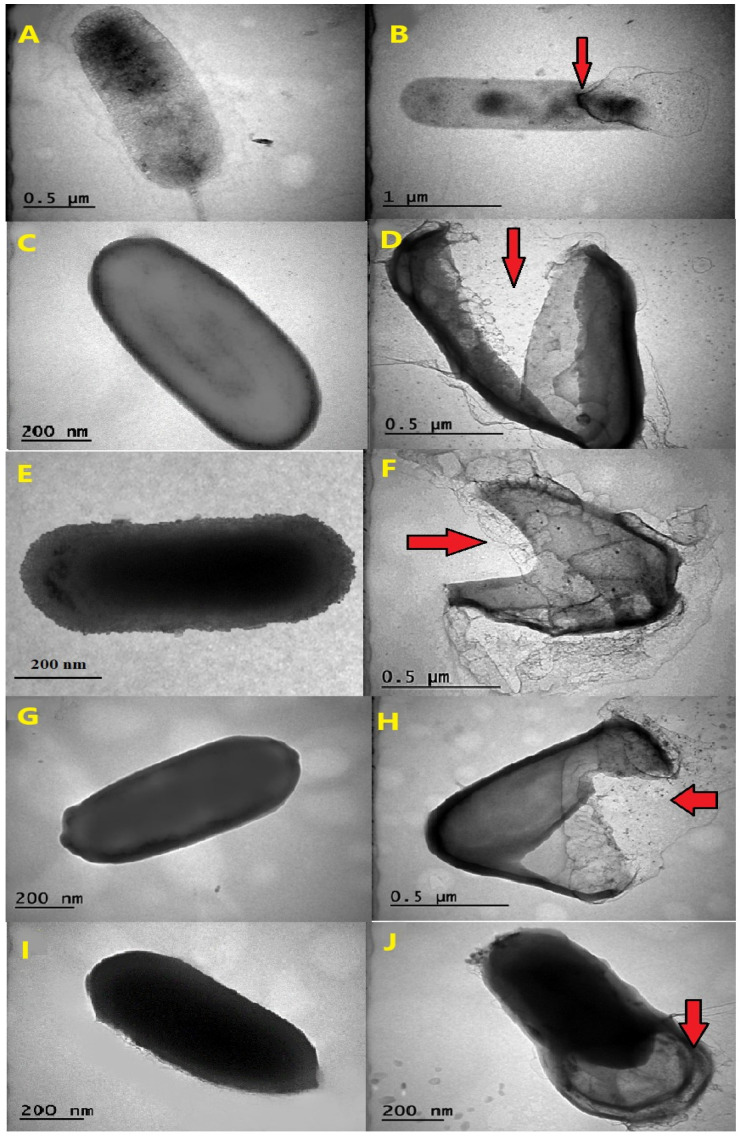
The effects AgNPs on the morphology of thermophilic bacteria. TEM images are shown of the untreated cells of *G. vulcani* 2Cx (**A**), *A. ayderensis* FMB1(**C**), *B. paralicheniformis* FMB2-1 (**E**), *B. licheniformis* 3CA (**G**), and *P. macerans* 3CA1 (**I**), and images are displayed for each strain after exposure to various AgNP concentrations (**B**,**D**,**F**,**H**,**J**). Red arrows indicate cellular damage.

The observed effects of AgNPs on thermophilic bacterial strains, as visualized through transmission electron microscopy (TEM), varied in terms of their destructive potential (Figure 2A–J). The images showed that the thermophilic strains grown in the absence of AgNPs mostly had undamaged and intact structures, while AgNP-treated cells were broken and destructed with many fragmented cell envelopes. The leakage of cytoplasmic content from inside the cells was observed for certain species, notably for *G. vulcani* 2Cx, and *P. macerans* 3CA1, providing clear evidence of cellular damage (Figure 2B,J).

The transmission electron micrographs of the AgNP-treated and untreated biomass of all the thermophilic bacterial strains clearly showed that AgNPs interacted with bacterial cells and thus had severe inhibitory effects that caused structural damage in the cell walls of the thermophilic bacteria (Figure 2A–J).

TEM analysis conducted in a previous study provided evidence that the exposure of *E. coli* cells to AgNPs results in the rapid and complete disruption of the cell membrane within a few minutes. The interaction between AgNPs and the thiol groups of proteins in the cell wall causes irreversible structural changes, resulting in the disruption of the cell wall and the formation of multiple pits at the sites affected by AgNPs [64].

### 3.5. Analysis of AgNP-Treated Bacterial Biomass Using FT-IR

**Figure 3:**
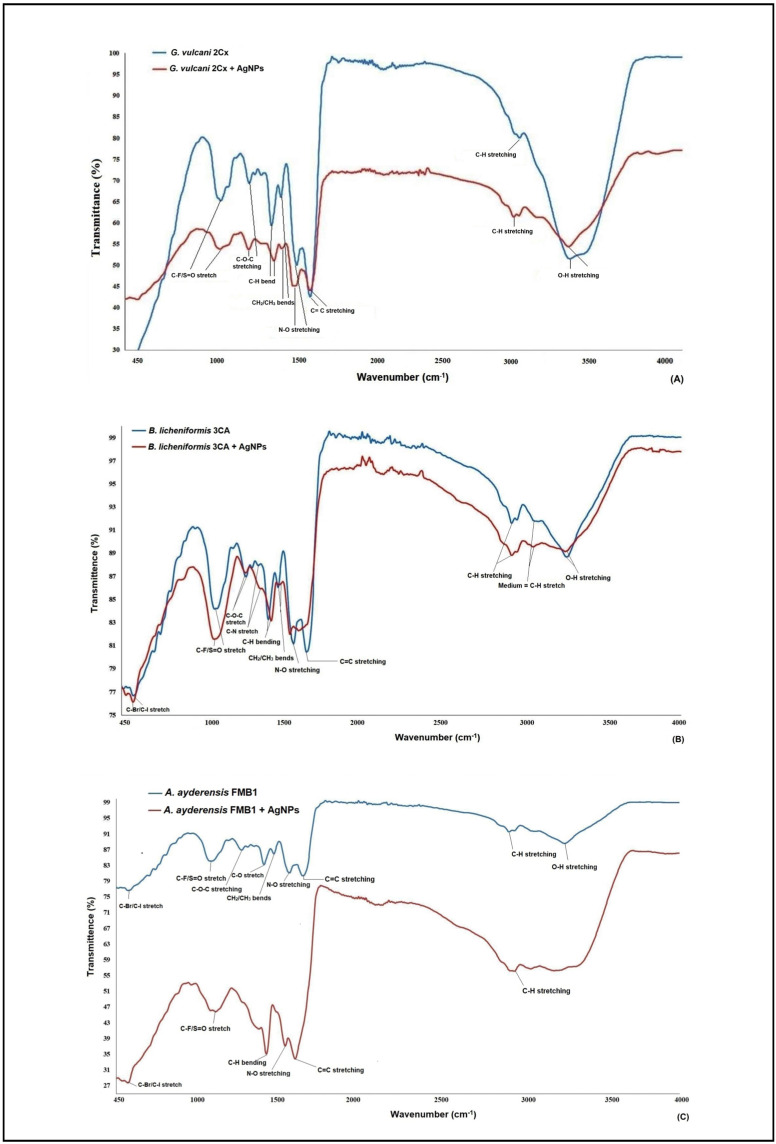
FT-IR (fourier transform infrared spectroscopy) data of both the AgNP-treated (500 μg mL^−1^) and untreated bacterial biomasses from *G. vulcani* 2Cx (**A**), *B. licheniformis* 3CA (**B**), and *A. ayderensis* FMB1 (**C**) exposed to 24 h incubation.

Figure 3A–C illustrate the FT-IR (fourier transform infrared spectroscopy) data of the AgNPs obtained from the selected three strains of *G. vulcani* 2Cx, *B. licheniformis* 3CA, and *A. ayderensis* FMB1. Significant changes were observed in the spectra, specifically in the peaks corresponding to different functional groups present on the surface of bacterial cells. Significant changes were observed in the FT-IR spectrum of the AgNP-treated bacterial cell biomass, with notable alterations being characterized by the narrowing and shifting of peaks. On average, the presence of carbon-related components, lipids, DNA, and proteins as well as amino-related components, polysaccharides, and other aromatic organic compounds were indicated as the most frequent biomolecules by the IR signals in both the control and AgNP-treated samples for all bacterial strains [65,66,67,68].

FT-IR analysis was used for evaluating significant variations in various functional groups of the most frequent biomolecules present on the cell surface of bacteria after treatment with different nanoparticles. For instance, silver and zinc nanoparticles were discovered to induce slight modifications in the functional groups of soil bacteria, such as *Azotobacter chroococcum* and *Pseudomonas mosselii*, as compared with those in the control group. This observation suggests that the inhibitory effects of nanoparticles on bacterial cells may lead to structural damage, especially to the cell membrane, thereby causing disruptions in the biochemical composition of the cells [63]. In a separate investigation, FT-IR was employed to elucidate the binding of fullerenol, which is a carbon nanostructure measuring 1 nm, to the lipid bilayer structure of a model bacterial cell. The analysis revealed that the OH groups present in the lipid layers were identified as the most active functional group during this binding process [69].

The analysis of the xenobiotic interactions with the biomolecule functional groups of cells by FT-IR is well documented for the α-helix protein (with the spectral band displayed at 1655–1658 cm^−1^), membrane lipids (at 2854 cm^−1^ and 2924 cm^−1^), and nucleic acids (at 1124 cm^−1^ and 1082 cm^−1^) [70,71,72,73]. The results obtained from the FT-IR spectra in the present study revealed notable changes to be observed through the narrowing and shifting of peaks, thereby indicating interactions of AgNPs with the cell walls, protein, enzymes, and the DNA in thermophilic bacteria. These are in agreement with our results also obtained with MIC, MBD, and TEM images; the generation of ROS; and the inhibition of enzyme biosynthesis and secretion.

### 3.6. Evaluating Cellular Viability in the Presence of AgNP Stress

**Figure 4:**
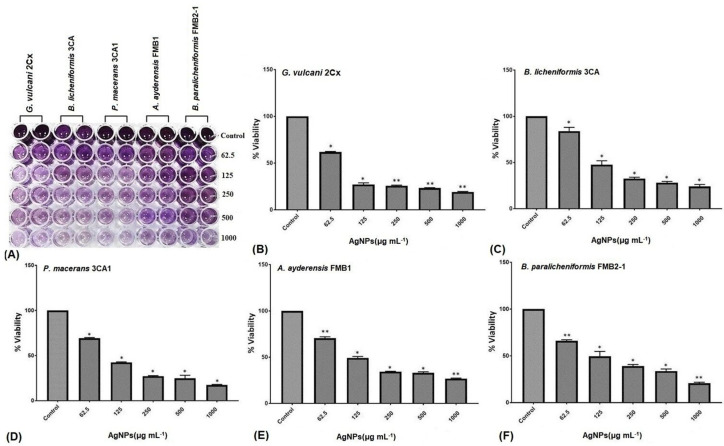
Inhibition of the cellular viability of *G. vulcani* 2Cx (**B**), *B. licheniformis* 3CA (**C**), *P. macerans* 3CA1 (**D**), *A. ayderensis* FMB1 (**E**), and *B. paralicheniformis* FMB2-1 (**F**) exposed to AgNP concentrations of 62.5–1000 μg mL^−1^ including the control. (**A**) The diminishing intensity of the purple color observed in the 96-well plates indicates a decrease in the metabolic activity of the bacterial cells. Asterisks represent the significant difference at * *p* ≤ 0.02 and ** *p* ≤ 0.0076.

The MTT assay finds extensive applications in the field of microbiology, primarily for the spectrophotometric evaluation of the metabolic activity exhibited by microorganisms [74]. Confirmation of cellular metabolism related to dehydrogenase activity was achieved by the formation of a distinct purple color, with the control group displaying the highest cellular activity and exhibiting the darkest coloration. The metabolic activity loss of the bacterial cells is represented by a decrease in color intensity shown in the microtiter wells (Figure 4A). The metabolic activity of all five bacterial strains, as indicated by the cell viability percentage (%), demonstrated a substantial reduction with increasing concentrations of AgNPs, i.e., those ranging from 62.5 μg mL^−1^ to 1000 μg mL^−1^ (Figure 4B–F). At a concentration of 62.5 μg mL^−1^ of AgNPs, *G. vulcani* 2Cx exhibited the lowest cell viability, with a recorded value of 61.87% (Figure 4B). In contrast, *B. licheniformis* 3CA demonstrated the highest cell viability at this concentration, with a remarkable value of 83.96% (Figure 4C). Furthermore, at a 125 μg mL^−1^ concentration, *G. vulcani* 2Cx showed the lowest viability (27.07%) (Figure 4B); however, no noteworthy variations were observed for the rest of the bacterial strains at this concentration (Figure 4C–F). On the other hand, at 250 μg mL^−1^, the lowest viability (25.75%) was observed for *G. vulcani* 2Cx while the highest cell viability (39.02%) was found in the *B. paralicheniformis* FMB2-1 bacterial strain (Figure 4B,F). At the highest AgNP concentration (1000 μg mL^−1^), *P. macerans* 3CA1 exhibited the lowest cell viability of 17.86% (Figure 4D).

Supporting results were also obtained from a previous study that utilized the TTC assay to evaluate the cell viability of several soil bacteria, including *B. thuringiensis, P. mosselii, S. meliloti,* and *A. chroococcum*, under the influence of silver and zinc oxide nanoparticles (62.5–1000 μg mL^−1^). The study assessed cellular respiration, particularly dehydrogenase activity, and the results indicated a significant decrease in cellular respiration when bacteria were exposed to the highest concentrations of both AgNPs and ZnONPs [63].

### 3.7. Quantifying Superoxide Generation in Bacterial Cells under AgNP-Induced Stress

**Figure 5:**
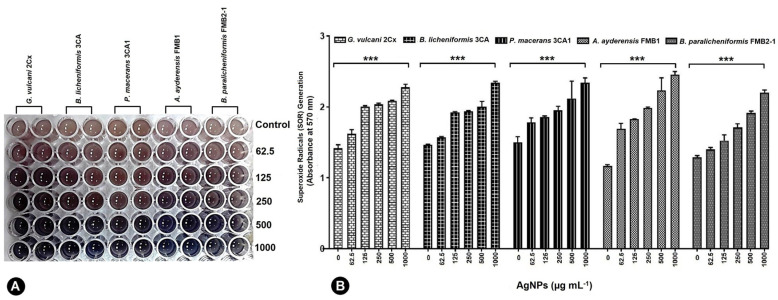
Generation of superoxide radicals (SORs) by *G. vulcani* 2Cx, *B. licheniformis* 3CA, *P. macerans* 3CA1, *A. ayderensis* FMB1, and *B. paralicheniformis* FMB2-1 exposed to the AgNP concentrations of 62.5–1000 μg mL^−1^ including the control (**B**). (**A**) The presence of a blue-colored formazan, which developed intracellularly in the 96-well plates, signifies an augmented production of SORs. Asterisks indicate the significant difference at *** *p* ≤ 0.0004.

Under the influence of 62.5–1000 μg mL^−1^ AgNPs, all five bacterial strains exhibited the production of superoxide radicals (SOR; O_2_^−^), which subsequently facilitated the reduction of nitro blue tetrazolium (NBT) to formazan. The resulting formazan formation was quantified using spectrophotometric methods (Figure 5A). *G. vulcani* 2Cx exhibited the maximum production of SOR radicals at AgNP concentrations of 125 and 250 μg mL^−1^ by the absorbance rate of 2.01 and 2.04 correspondingly (Figure 5B).

For the control and AgNP concentration of 62.5 μg mL^−1^, the maximum SOR production was observed for *P. macerans* 3CA1 with significant absorbance values of 1.50 and 1.78, respectively (Figure 5B). On the other hand, *A. ayderensis* FMB1 demonstrated the highest production of SORs r at AgNP concentrations of 500 and 1000 μg mL^−1^ with the corresponding absorbance values of 2.23 and 2.26, while the lowest production was obtained in the untreated control group (1.17) (Figure 5B).

Reactive oxygen species (ROS) occur due to incomplete oxygen reduction within different metabolic pathways, serving as by-products in these processes. Their presence at low concentrations is indispensable for maintaining cellular functionality, and their concentration within the cellular system is intricately regulated by the intricate antioxidant defense system [75]. Ensuring that intracellular ROS production remains within the physiological range is advantageous for cellular health and functioning. However, elevated levels of intracellular ROS can trigger apoptosis, which is a process of programmed cell death [76].

Moreover, the exposure of silver NPs to human cells induces genotoxicity, cytotoxicity, and inflammation in a cell type-dependent manner. The oxidative stress-dependent toxicity of AgNPs has also been well documented in animals [77,78]. Ahmed et al. [63] found that SOR production increased with an increasing concentration of metal NPs in beneficial soil bacteria. The elevation of cellular oxidative stress in microorganisms has been an important antibacterial mechanism of metallic NPs and heavy metals ions such as Ag^+^. The generation of reactive oxygen species (ROS) and free radical species is responsible for the potent antiviral, antifungal, and antibacterial activity exhibited by AgNPs. ROS include hydrogen peroxide (H_2_O_2_), singlet oxygen (O_2_), superoxide radical (O_2_^−^), hydroxyl radical (OH^•^), and hypochlorous acid (HOCl). The AgNP treatment of bacterial cells leads to ROS production and subsequent oxidative stress, which induces cell death, possibly through the hyperoxidation of proteins, lipids, and DNA [79,80].

### 3.8. Effect of Silver NPs on Enzyme Biosynthesis

**Figure 6:**
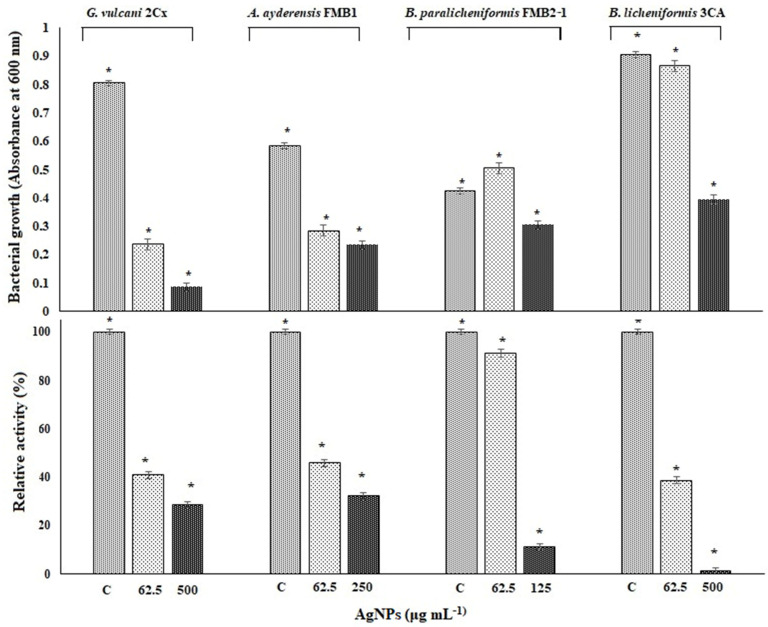
The effects of AgNPs on bacterial growth and levels of β-galactosidase biosynthesis in tested thermophilic bacteria. The upper chart shows the bacterial growth inhibition of *G. vulcani* 2Cx, *A. ayderensis* FMB1, *B. paralicheniformis* FMB2-1, and *B. licheniformis* 3CA exposed to specified AgNP concentrations of 62.5, 125, 250, or 500 μg mL^−1^. The lower chart indicates the inhibition of β-galactosidase biosynthesis expressed by these strains exposed to the same concentrations of AgNPs during 24 h incubation. Absorbance of *o*-nitrophenol (yellow-colored end product of the substrate ONPG) measured at 405 nm obtained for controls (taken as 100% relative activity) is plotted against absorbances obtained for treated samples. Values are the mean of three independent experiments ± SD. Asterisks indicate the significant difference at * *p* < 0.001; C indicates control.

In order to determine the inhibition of β-galactosidase biosynthesis in bacterial cells, only four thermophilic bacteria strains in the presence of sub-MICs of AgNPs between 62.5 and 500 μg mL^−1^ were grown in a liquid BM for 24 h in a shaking incubator at 50 °C. *P. macerans* 3CA1 was excluded here because growth was not consistent throughout the experiment. Figure 6 shows both the inhibition of bacterial growth and β-galactosidase biosynthesis in the tested thermophilic bacteria exposed to the specified AgNP concentrations in the same experiment set. After a 24 h exposure, the β-galactosidase activity of *G. vulcani* 2Cx decreased to 41% at 62.5 μg mL^−1^ AgNP and further decreased to 28.8% at 500 μg mL^−1^ compared with the untreated controls with 100% relative activity. Likewise, for *A. ayderensis* FMB1, the activity decreased to 45.9% at 62.5 μg mL^−1^ AgNPs and 32.2% at 250 μg mL^−1^, whereas for *B. licheniformis* 3CA, the activity decreased to 38.8% at 62.5 μg mL^−1^ AgNPs, potentially reaching complete inhibition. In addition, for *B. paralicheniformis* FMB2-1, it was observed that the activity was not affected much at 91.2% in the presence of 62.5 μg mL^−1^ AgNPs but decreased sharply to 11.2% at a AgNP concentration of 125 μg mL^−1^ (Figure 6).

*β*-Galactosidase, which is also known as a glycoside hydrolase enzyme, hydrolyzes galactopyranosides such as lactose and produces galactooligosaccharides by catalyzing the trans-galactosylation reaction. Recent studies have focused on thermostable β-galactosidases obtained from thermophiles. There are studies on beta-galactosidase obtained from thermophilic bacteria such as *Alicyclobacillus acidocaldarius* subsp. rittmannii [47,48], *Bacillus licheniformis* KG9 [49], *Anoxybacillus* sp. KP1 [50], *A. ayderensis* [81], *Anoxybacillus* sp. FMB1 [82], *Anoxybacillus* sp. AH1 [83], *B. subtilis* 4NK, and *B. paralicheniformis* 5NK [51]. Thermophilic *β*-galactosidases are used in many biotechnological fields, such as in medical settings to address lactose intolerance, in the food industry to prevent the crystallization of lactose in dairy products, and in environmental contexts to mitigate water pollution stemming from whey.

Notably, sodium dodecyl sulfate (SDS) is employed as a membrane permeabilizer to facilitate the uptake of *o*NPG, a substrate typically impermeable to the cytoplasmic membrane, into bacterial cells, which is crucial for accurately quantifying the β-galactosidase activities exhibited by the tested bacterial strains. SDS plays a vital role in enhancing the penetration of *o*NPG into the cytoplasm, enabling the reliable measurement of β-galactosidase enzyme activity. In a previous study, we demonstrated that heavy metals alone significantly inhibited the biosynthesis of α-amylase and β-galactosidase in both *E. coli* and *B. subtilis* [56].

**Figure 7:**
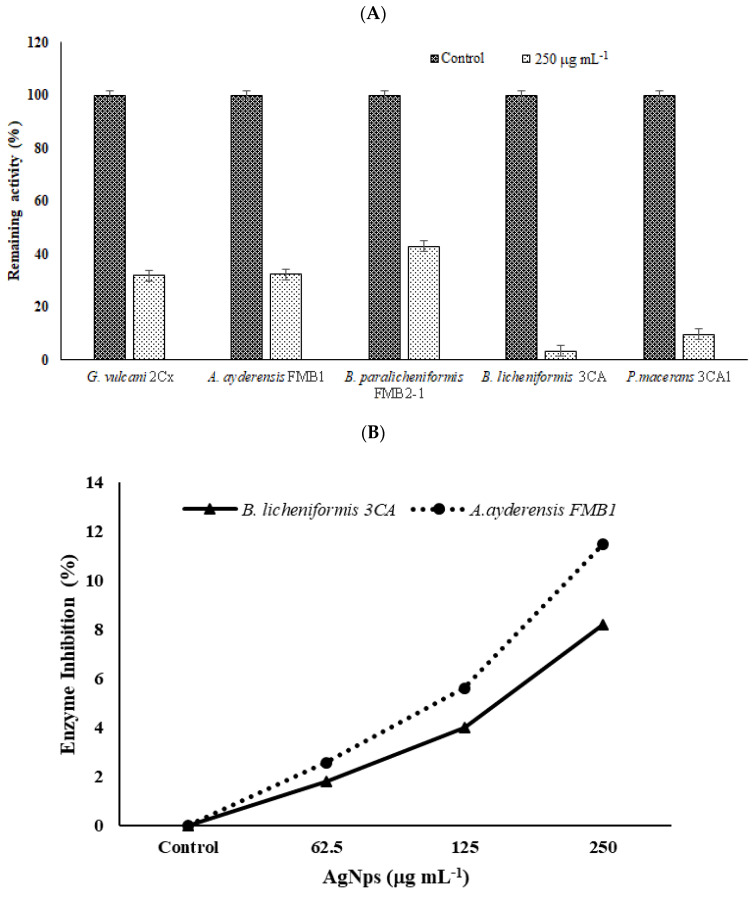
The effects of AgNPs on β-galactosidase secretion and crude enzyme inhibition. (**A**) Inhibition of enzyme secretion by bacterial strains *G. vulcani* 2Cx, *A. ayderensis* FMB1, *B. paralicheniformis* FMB2-1, *B. licheniformis* 3CA, and *P. macerans* 3CA1. Each was exposed to a concentration of 250 μg mL^−1^. (**B**) Inhibition of crude β-galactosidases of *A. ayderensis* FMB1 and *B. licheniformis* 3CA exposed to 62.5, 125, and 250 μg mL^−1^ sub-MICs of AgNP. Absorbances of *o*-nitrophenol measured at 405 nm obtained for controls (taken as 100% relative activity) are plotted against absorbances obtained for the treated samples. Values are the mean of three independent replicates ± SD.

Substantial inhibition of β-galactosidase biosynthesis and its extracellular secretion in the thermophilic bacteria (please see Figure 6 and Figure 7) may result from the entrance of AgNPs into the bacterial cell, which bind to cellular structures and biomolecules such as DNA, lipids, and proteins, thus causing lethal toxic effects in microorganisms. In particular, the interaction of AgNPs with ribosomes induces their denaturation, leading to the inhibition of translation and protein synthesis. This disruption of ribosomal function hinders the crucial processes involved in protein production, causing a notable reduction in protein synthesis [14,84,85]. AgNPs are also found to bind and inactivate the cytoplasmic proteins needed for the production of ATP, thus disrupting cellular functions. Additionally, AgNPs interact with disulfide bonds and block the active sites, resulting in the inactivation of the enzymes and proteins that are associated with the cell membrane [86]. AgNPs have also been observed interacting with the carboxyl and thiol groups of β-galactosidase, resulting in the inhibition of critical intracellular processes and ultimately culminating in cell death [87]. Protein synthesis is also inhibited by Ag ions that prevent tRNA from binding with the ribosome subunit [88]. Ionic silver (Ag^+^) is known for its affinity in interacting with -SH groups found in proteins and enzymes, resulting in conformational changes in their tertiary structure. This interaction disrupts the proper binding of substrates to their respective active sites, thereby affecting the functional activity of these biomolecules [89].

### 3.9. Effect of Silver NPs on Enzyme Secretion and Inhibition

In order to determine the inhibition effect of β-galactosidase secretion to the outside of bacterial cells, bacteria grown in a BM containing 2% lactose were treated with a sub-MIC of AgNPs (250 µg mL^−1^), and the extracellular enzyme activities in supernatants were measured after 4 h incubation. Samples without any added AgNPs were used as the controls. Figure 7A shows the effect of AgNPs on enzyme secretion by bacterial cells. It is seen that compared with the untreated controls (100% relative activity), in the presence of 250 µg mL^−1^ AgNP, the enzyme secretion by thermophilic bacteria was reduced to 31.9% for *G. vulcani* 2Cx, 32.4% for *A. ayderensis* FMB1, 42.9% for *B. paralicheniformis* FMB2-1, 3.3% for *B. licheniformis* 3CA, and 9.7% for *P. macerans* 3CA1.

In Figure 7B, the graph depicts the inhibition of extracellular enzymes in samples subjected to different sub-MICs of AgNPs. Comparatively, in comparison with the untreated control, the inhibition of crude enzyme activity in all the tested AgNP concentrations was not considerable, being only 2.6% and 11.5% for the enzyme of *A. ayderensis* FMB1 in the presence of 62.5 µg mL^−1^ and 250 µg mL^−1^ AgNPs, respectively, while the inhibition was 1.7% and 8.2% for *B. licheniformis* 3CA, respectively.

The exact mechanism underlying the antimicrobial effects of metal nanoparticles is still not fully comprehended. However, numerous studies have provided evidence for the lethal effect and growth inhibition of bacteria resulting from the destructive impact of AgNPs. These effects are attributed to the direct interaction between AgNPs and biomolecules on the bacterial surface through electrostatic attraction. This interaction leads to various consequences, including alterations in cell morphology, the penetration of AgNPs into the cytoplasm alongside ions, increased membrane permeability due to membrane damage, cytoplasmic leakage, and the generation of intracellular oxidative stress in the form of superoxide anions. This oxidative stress progressively damages cellular constituents and membranes, disrupts the electron transport chain, halts the respiratory process, and causes the destruction of enzyme activity and the denaturation of proteins. Ultimately, these combined effects result in the eventual death of the bacterial cells [90,91].

## 4. Conclusions

The present study emphasizes the toxic effects of AgNPs on morphological, physiological, and biochemiacal aspects including enzyme biosynthesis and secretion in thermophilic bacteria. Although five thermophilic strains exposed to AgNPs had a growth decrease in the number of CFU mL^−1^, *G. vulcani* 2Cx and *B. paralicheniformis* FMB2-1 were more sensitive to AgNPs. AgNPs at all the tested concentrations exhibited severe structural damage on the thermophilic bacteria, causing cytoplasmic leakage as well as broken and destructed cells. There were also a significant reduction in cell viabilities and enhanced superoxide radical (SOR) generation consistent with an increasing dose rate of AgNPs. Conclusively, AgNP treatments caused a significant inhibition of thermostable β-galactosidase biosynthesis and extracellular secretion by potent thermophilic strains that are likely to be used in various areas of industry. The mechanisms of the antimicrobial properties and toxicological effects of metallic NPs are not fully understood yet and need to be focused on in future work.

## Figures and Tables

**Table 1:** Characteristics of silver (Ag) nanoparticles.

AgNP Characteristics	
Purity\	99.5% (metal basis)
Average particle size	35 nm
Specific surface area	20–30 m^2^/g
Particle morphology	Spherical
Appearance	Gray powder

**Table 2:** Morphological and physiological features of the strains.

Bacterial Strains	Shape	Spore Forming	Pigmentation	Optimum Temperature (°C)	Optimum pH	Reference
*G. vulcani* 2Cx	rod	+	light yellow	55	7.0	[53]
*B. licheniformis* 3CA	rod	+	creamy white	55	8.0	[53]
*P. macerans* 3CA1	rod	+	creamy white	50	7.0	[53]
*A. ayderensis* FMB1	rod	+	yellow/orange	50	7.0	[54]
*B. paralicheniformis* FMB2-1	rod	+	creamy white	50	7.0	[55]

**Table 3:** MIC and MBC of AgNPs against thermophilic bacteria.

Bacterial Strains	AgNPs (μg mL^−1^)	
	MIC	MBC
*G. vulcani* 2Cx	500	1000
*B. licheniformis* 3CA	1000	1500
*P. macerans* 3CA1	500	1000
*A. ayderensis* FMB1	1000	1500
*B. paralicheniformis* FMB2-1	500	1000

## Data Availability

All data supporting the reported results can be obtained from the corresponding author.
